# Association of ethylene oxide exposure with serum neurofilament light chain levels among American adults

**DOI:** 10.3389/fpubh.2025.1545164

**Published:** 2025-03-12

**Authors:** Xiuwen Yang, Huaili Feng, Ting You, Zhaoyi Liu, Fanwei Sun, Chengzhi Chen, Jingfu Qiu

**Affiliations:** ^1^Research Center for Environment and Human Health, School of Public Health, Chongqing Medical University, Chongqing, China; ^2^Faculty of Medicine, The Chinese University of Hong Kong, Shatin, Hong Kong SAR, China

**Keywords:** NHANES, ethylene oxide, neurofilament light chain, neurotoxicity, adults

## Abstract

**Objective:**

To explore the relationship between Ethylene oxide (EO) expousure and serum neurofilament light chain (NfL).

**Method:**

A data of 559 adults from the 2013–2014 National Health and Nutrition Examination Survey (NHANES) was analyzed, and the relationship between log-transformed EO hemoglobin adducts (HbEO) and serum NfL levels was assessed using multiple linear regression models and restricted cubic spline functions. Stratified analysis was conducted to explore the correlations within different subgroups. Mediation analysis was employed to investigate potential mediating factors.

**Results:**

The higher HbEO levels were consistently associated with elevated serum NfL concentrations among the study participants (*β* = 0.07, 95%CI: 0.00–0.14; *p* = 0.044), and serum NfL levels increased with rising HbEO levels (*p* for trend = 0.013). The restricted cubic spline results confirmed the linear relationship between serum NfL and HbEO. Subgroup analysis indicated a significant positive correlation, particularly among non-Hispanic white people, individuals aged 40–59, and heavy drinkers.

**Conclusion:**

These findings highlighted the neurotoxic potential of EO and underscored the importance of monitoring EO exposure to mitigate its adverse health effects.

## 1 Introduction

EO is a chemical widely used in industry, primarily as a sterilizing agent and chemical intermediate (1). Despite its broad applications, EO is considered a potent mutagen and carcinogen, raising significant public health concerns (2). The International Agency for Research on Cancer (IARC) classified EO as a Group 1 carcinogen, indicating that there is sufficient evidence of its carcinogenicity in humans (39). Human exposure to EO occurs through both endogenous and exogenous pathways. Endogenously, EO is produced from ethylene through multiple metabolic processes, resulting in baseline exposure levels in all individuals. Exogenous sources include cigarette smoke, incomplete fossil fuel combustion, and natural processes such as forest fires and volcanic eruptions (3, 4). Once inhaled, EO is rapidly absorbed through the respiratory tract and distributed throughout the body via systemic circulation (4).

Recent epidemiological studies have linked EO exposure to various adverse health outcomes, including kidney stones (5), asthma (6), diabetes (7), hypertension (8) and dyslipidemia (9). Notably, emerging evidence suggests potential neurotoxic effects of EO, with studies reporting associations between EO exposure and neurological symptoms, particularly depression (10, 11). However, the underlying mechanisms of EO’s neurotoxicity and its impact on specific biomarkers of neural damage remain poorly understood.

NfL is a protein found in the axons of neurons and is a crucial component of the neuronal cytoskeleton (12). Elevated serum NfL levels was considered a sensitive and specific biomarker for neuronal damage and neurodegenerative diseases (13). In recent years, serum NfL has gained attention for its utility in diagnosing and monitoring various neurological disorders, including multiple sclerosis (14), Alzheimer’s disease (15), and traumatic brain injury (16). Measuring serum NfL levels provided a non-invasive means to assess neural health and has become an important tool in epidemiological research. Recent studies have demonstrated that exposure to environmental pollutants, such as volatile organic compounds (VOCs) (17) and glyphosate (18), can influence serum NfL levels. These findings suggested its potential utility as a biomarker for environmental neurotoxicity.

Given the increasing evidence of EO’s harmful effects on the nervous system and the established role of NfL as a biomarker for neuronal damage, we hypothesized that EO exposure might be associated with elevated serum NfL levels. To test this hypothesis, we analyzed data from the 2013–2014 National Health and Nutrition Examination Survey (NHANES) to assess the relationship between EO exposure [measured by HbEO (19)] and serum NfL levels among American adults. Additionally, we investigated potential mediating roles of metabolic factors, including blood pressure, blood glucose, lipid metabolism, and body mass index in this association. The findings of this study could provide critical insights into the neurotoxic mechanisms of EO and inform public health policies aimed at mitigating its adverse health impacts.

## 2 Materials and methods

### 2.1 Study population

**Figure 1:**
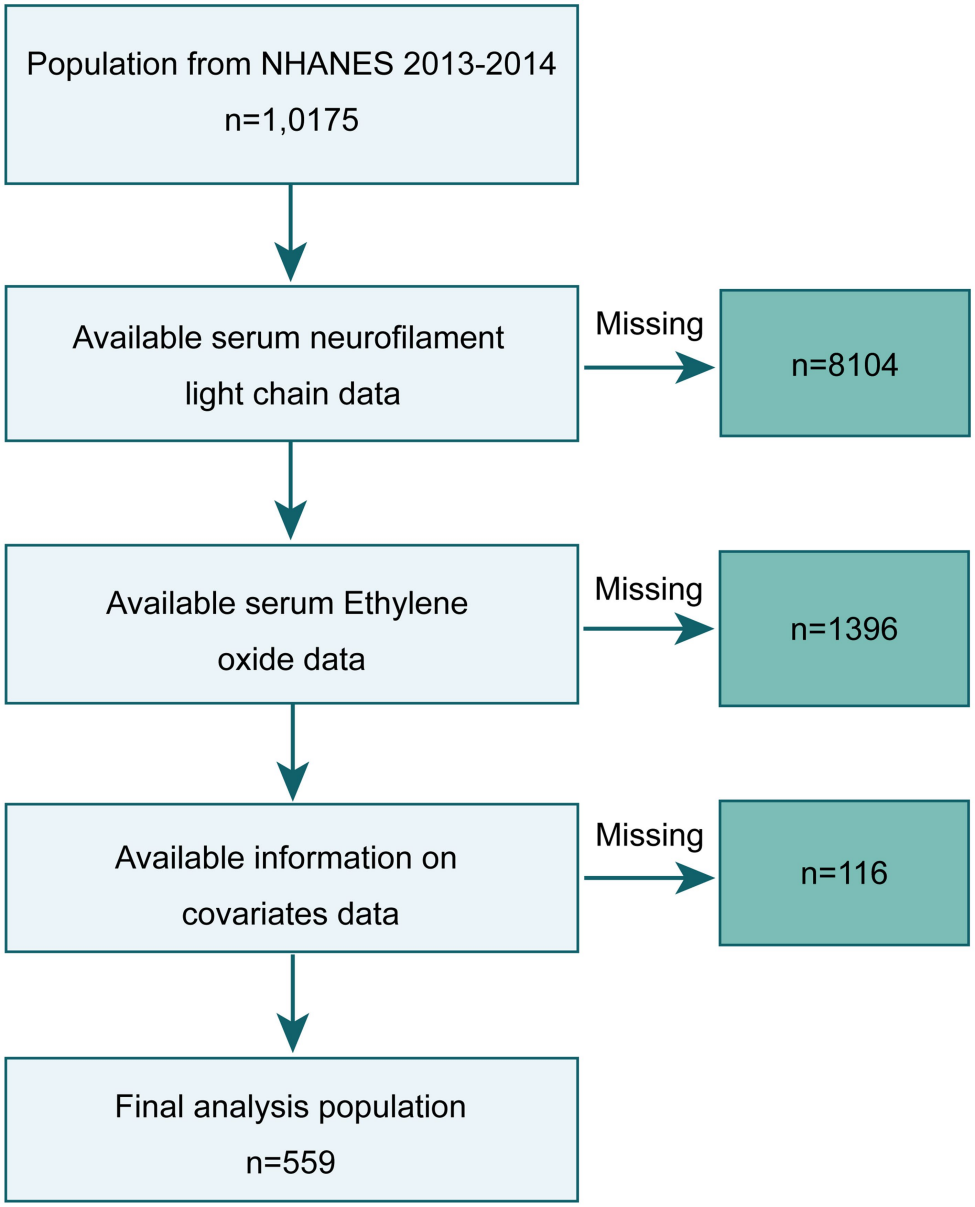
Flow chart of the inclusion of the study population.

NHANES is an ongoing cross-sectional survey conducted by the National Center for Health Statistics (NCHS) within the Centers for Disease Control and Prevention (CDC). NHANES has received ethical approval from the NCHS Ethics Review Board, and all study participants provided informed consent. We selected data from the NHANES 2013–2014 survey for cross-sectional analysis, knowing that these data included serum NfL measurements and HbEO measurements in a subsample of participants aged 6 years and older. Out of 10,175 participants in 2013–2014, we initially excluded 9,500 individuals lacking serum NfL and HbEO data and 116 individuals missing other covariate information (annual household income: 29, blood lipids: 16, body mass index (BMI): 5, depression information: 66), resulting in a final sample of 559 subjects. The study inclusion process is illustrated in Figure 1.

### 2.2 Detection of HbEO

Due to the longer half-life of HbEO compared to EO in the humanbody, NHANES recommends using HbEO to assess EO exposure. Briefly, red blood cell (RBC) samples were washed, packed, processed, and shipped to the National Center for Environmental Health for testing. Then, total hemoglobin levels were measured and identified, and hemoglobin adducts were estimated. Hb levels modified by the Edman reaction were measured using a commercial test kit (Tech Diagnostics, Anaheim, CA). HbEO levels in human whole blood or RBCs were determined using high-performance liquid chromatography–tandem mass spectrometry (HPLC-MS/MS). Detailed experimental procedures can be found in the NHANES Laboratory/Medical Technologists Procedures Manual^1^. All test results met the quality control and assurance standards of the NCEH Laboratory Sciences Division.

### 2.3 Measurement of serum NfL

Serum NfL levels were measured using the fully automated Attelicaimmunoassay system developed by Siemens Healthcare, using acridinium ester chemiluminescence and paramagnetic particles for quantification. The detection rate for serum NfL was 98.4%. For concentrations below the limit of detection (LLOD, 3.9 pg/mL), an estimated value (LLOD divided by the square root of 2) was used. For detailed information on the research methods, visit: https://wwwn.cdc.gov/Nchs/Nhanes/2013-2014/SSSNFL_H.htm.

### 2.4 Covariates

**Figure 2:**
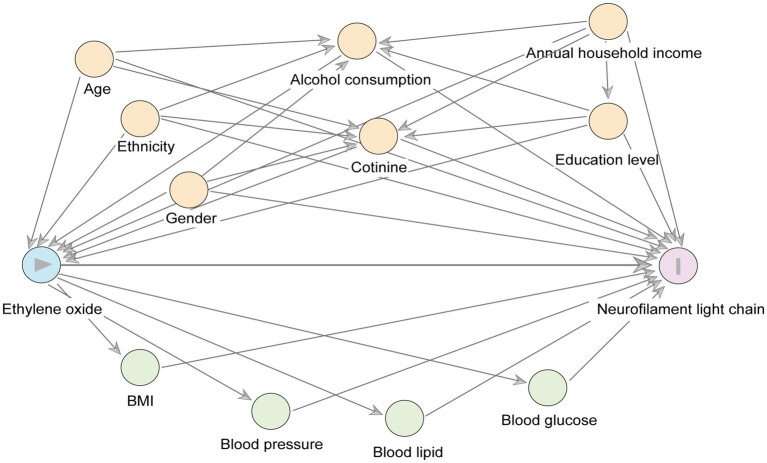
A directed acyclic graph (DAG) represents potential covariates and mediating variables in the relationship between ln-HbEO (exposure) and ln-sNfL (outcome). Yellow circles represent the common ancestry variable (i. e., confounders) between the exposure variables and the outcome variables, and green circles represent the potential mediating variables.

We used a directed acyclic graph (DAGs) to preselect covariates as potential confounders. As shown in Figure 2, which displayed the main relationships between exposure, outcome, confounders, and mediators. We included age, sex, race, household income (≤$25,000, ≥$25,000) (17), education level (less than high school, high school, higher than high school), alcohol consumption (No: Drinking less than 12 drinks in the past year, moderate: Women average less than one drink per day or men average less than two drinks per day, severe: Women average more than one drink per day or men average more than two drinks per day) (20), and serum cotinine levels (<0.05, 0.05–2.99, ≥3.00) (21, 22). Depression was measured using the 9-item Patient Health Questionnaire (PHQ-9), with scores greater than 10 indicating depression (23). Additionally, to explore mediating factors between HbEO and serum NfL, we included measurements of blood pressure (systolic and diastolic), blood glucose (HbA1c, fasting glucose), blood lipids (HDL cholesterol, LDL cholesterol, total cholesterol, triglycerides), and body mass index (BMI).

### 2.5 Statistical analysis

We conducted all analyses according to the recommended NHANES analysis guidelines, including sample weights (WTSSNH2Y) and accounting for clustering and stratification. For more details on sample weights and other analytical considerations, refer to the “NHANES Analytic Guidelines” and the online NHANES tutorials at https://wwwn.cdc.gov/nchs/nhanes/analyticguidelines.aspx. Continuous variables are presented as means ± standard error (SE), and categorical variables as unweighted counts and weighted percentages [*N*(%)]. Categorical variables were analyzed using chi-square tests, and continuous variables using Student’s two-tailed *t*-tests or one-way ANOVA. Due to the right-skewed distribution of the data, we used natural logarithms of HbEO and serum NfL (ln-HbEO and ln-sNfL).

We assessed the relationship between ln-HbEO and ln-sNfL levels in the U.S. adult population using multiple linear regression models. We quantified these relationships using *β* coefficients and their corresponding 95% confidence intervals (95%CI). To test for trends, we converted ln-HbEO into quartiles and compared the β coefficients for each quartile to the lowest quartile (Q1). We performed tests for trends by entering the median value of ln-HbEO as a continuous variable in the models. Three models were constructed: a crude model without adjusting for any covariates, model 1 adjusted for age, sex, race, education level, and household income, and model 2 further adjusted for alcohol consumption and serum cotinine levels based on model 1. We identified potential multicollinearity by calculating the variance inflation factor (VIF). We conducted restricted cubic spline (RCS) analysis to characterize the non-linear relationship between ln-HbEO and ln-sNfL levels, guiding knot selection by minimizing the Akaike information criterion (AIC) and assessing non-linearity using the likelihood ratio test.

To elucidate the relationship between HbEO and serum NfL levels in different subgroups, we conducted additional stratified analyses. Finally, we used the R package MEDIATION to investigate whether blood pressure, blood glucose, blood lipids, and BMI mediated the relationship between HbEO and serum NfL, and whether serum NfL levels mediated the relationship between HbEO and depression. All statistical analyses were performed using R studio (R foundation, version 4.2.3). Significance was determined at a two-sided *p*-value threshold of <0.05.

### 2.6 Sensitivity analysis

We conducted two sensitivity analyses to assess the robustness of our findings. First, multiple linear regression analyses were performed using a larger sample (*n* = 641) that only excluded participants with missing covariates while retaining those with missing mediator variables. Second, unweighted analyses were conducted using the final dataset (*n* = 559) to examine whether the complex survey design and sampling weights substantially influenced our results.

## 3 Results

### 3.1 Baseline characteristics

Table 1 summarized the baseline characteristics of the study participants. Among the 559 subjects included, the geometric mean (SE) of HbEO was 29.37 (1.05), and the geometric mean of serum NfL was 11.94 (1.03). The study population had the highest proportion of Non-Hispanic white people (67%). Table 1 also showed the geometric means (SE) of HbEO and serum NfL across different subgroups. Men had higher levels of HbEO and serum NfL than women, individuals aged 60 and above had the highest serum NfL levels, and the Non-Hispanic white people group had the highest serum NfL levels. Additionally, significant differences in HbEO levels were observed across different races, BMI levels, and education levels. Heavy drinkers and individuals with higher serum cotinine levels also exhibited significantly higher HbEO levels.

**Table 1:** The geometric means (SE) of HbEO and serum NfL levels across different subgroups.

Characteristic	*N*	ln-HbEO (pmol/g Hb)	*p*-value	ln-sNfL (pg/mL)	*p*-value
Total	559	29.37 (1.05)		11.94 (1.03)	
Gender			0.016		0.003
Male	277 (51%)	32.79 (1.05)		13.20 (1.03)	
Female	282 (49%)	25.79 (1.04)		10.70 (1.03)	
Age (years)			0.4		<0.001
20–39	203 (39%)	32.46 (1.05)		8.76 (1.03)	
40–59	210 (38%)	29.37 (1.05)		12.06 (1.03)	
≧ 60	146 (23%)	24.05 (1.04)		20.29 (1.02)	
Ethnicity			0.009		<0.001
Non-Hispanic white people	268 (67%)	29.08 (1.05)		13.46 (1.03)	
Non-Hispanic black people	91 (11%)	46.53 (1.06)		10.07 (1.03)	
Mexican American	82 (10%)	20.09 (1.02)		7.92 (1.03)	
Other Hispanic	56 (6.3%)	28.79 (1.04)		9.12 (1.03)	
Others	62 (5.6%)	24.29 (1.04)		11.02 (1.02)	
BMI (kg/m^2^)			0.017		0.5
< 25	197 (35%)	35.87 (1.05)		11.82 (1.03)	
25 ~ < 30	163 (30%)	29.08 (1.05)		12.18 (1.02)	
≧ 30	199 (35%)	24.05 (1.04)		11.82 (1.03)	
Annual household income			0.088		>0.9
≦ 25,000	176 (22%)	38.09 (1.05)		12.18 (1.03)	
≧ 25,000	383 (78%)	27.11 (1.05)		11.82 (1.03)	
Education level			0.001		0.12
Less than high school	111 (14%)	49.90 (1.05)		11.59 (1.03)	
High school	117 (20%)	36.23 (1.05)		13.60 (1.03)	
Higher than high school	331 (66%)	24.53 (1.04)		11.47 (1.03)	
Alcohol consumption			0.042		0.005
No	133 (20%)	24.53 (1.04)		11.47 (1.03)	
Moderate	172 (32%)	25.03 (1.05)		13.46 (1.03)	
Heavy	254 (49%)	34.47 (1.05)		11.25 (1.03)	
Cotinine (ng/mL)			<0.001		0.4
< 0.05	292 (53%)	16.44 (1.02)		11.25 (1.03)	
0.05–2.99	101 (16%)	19.49 (1.03)		11.94 (1.03)	
≧ 3.00	166 (30%)	100.48 (1.05)		13.33 (1.03)	

Data are presented as *N*(%) or Mean ± SE.

Tested by student’s 2-tailed *t* test or by one-way analysis of variance.

### 3.2 Association between HbEO and serum NfL

**Figure 3:**
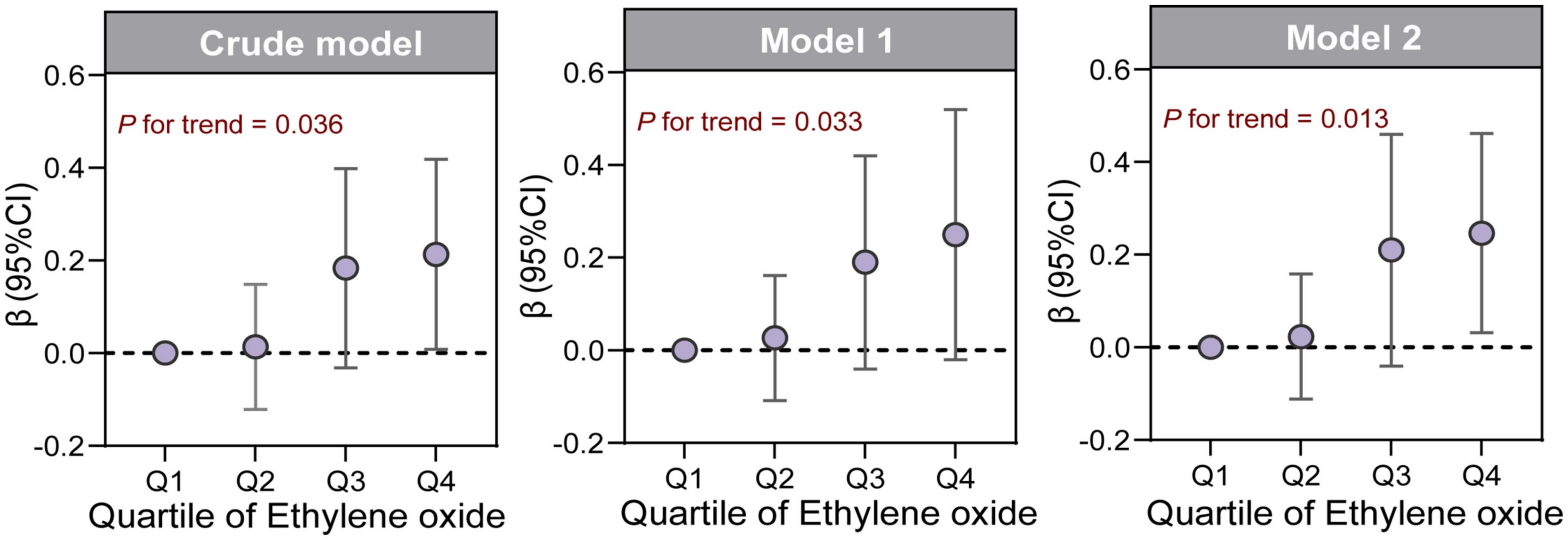
*β* (95%CI) of ln-sNfL across quartiles of ln-HbEO in multiple linear regression models, as well as the trend test.

**Figure 4:**
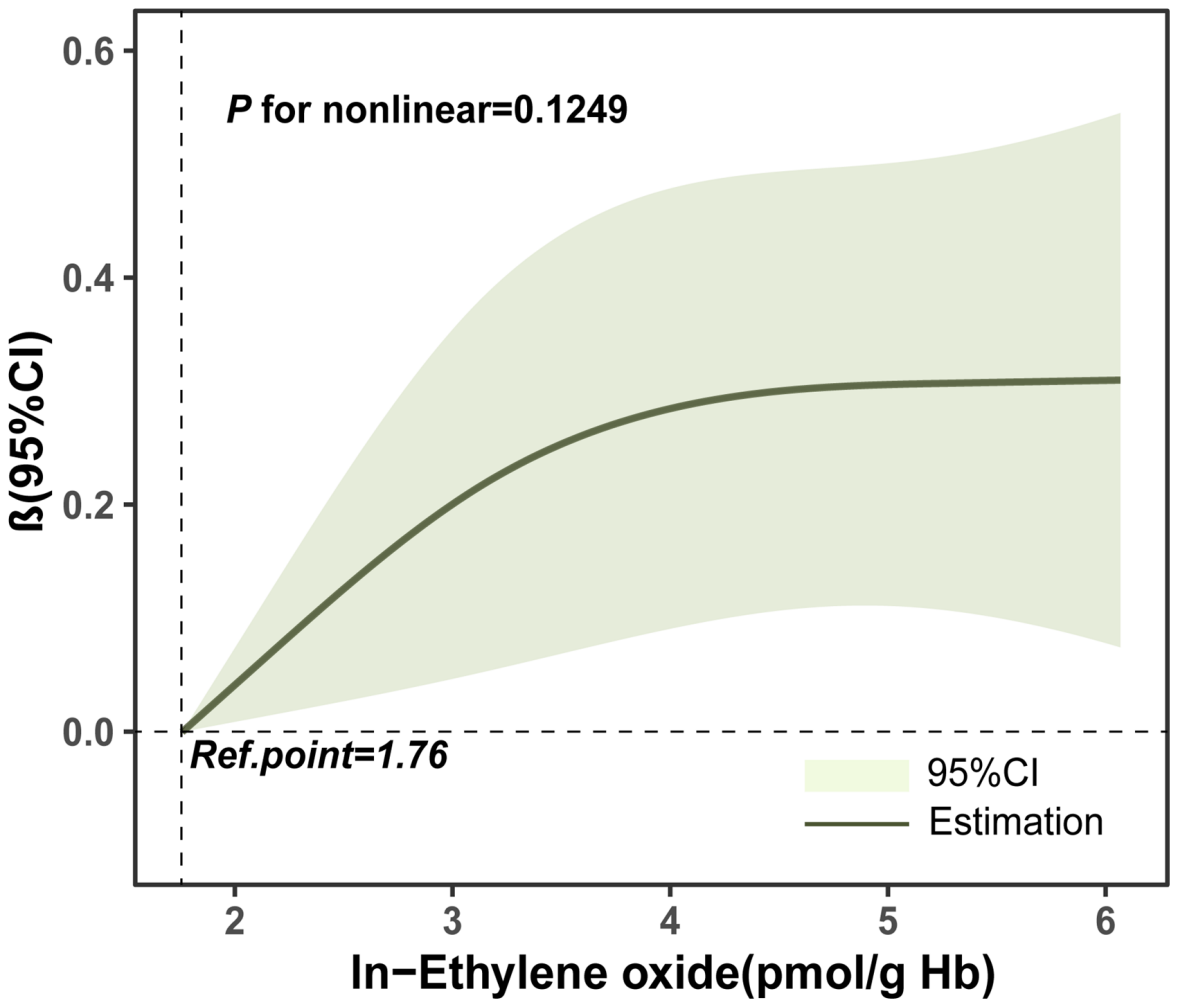
Restricted Cubic Spline (RCS) plots of the relationship between ln-HbEO and ln-sNfL. Adjusted for gender, age, ethnicity, annual household income, education level, alcohol consumption and serum cotinine.

We explored the relationship between ln-HbEO and ln-sNfL using multiple linear regression models. When ln-sNfL was included as a continuous variable in the regression models (Table 2), we found that a one-unit increase in ln-HbEO levels was positively associated with ln-sNfL in all models (Crude model: ß = 0.06, 95% CI: 0.00–0.12; SE = 0.027; *p* = 0.036. Model 1: ß = 0.08, 95% CI: 0.01–0.15; SE = 0.028; *p* = 0.032. Model 2: ß = 0.07, 95% CI: 0.00–0.14; SE = 0.025; *p* = 0.044). Next, we converted ln-sNfL into quartiles and used the lowest quartile (Q1) as the reference category to construct multiple linear regression models (Table 3). The results showed that after adjusting for all covariates, the highest quartile of ln-sNfL was associated with a 0.25-unit increase in ln-HbEO levels compared to the lowest quartile (ß = 0.25, 95% CI: 0.03–0.46; SE = 0.095; *p* = 0.038). Additionally, we observed that ln-sNfL levels increased with ln-HbEO levels in all models (Crude model: *p* for trend = 0.036; Model 1: *p* for trend = 0.033; Model 2: *p* for trend = 0.013) (Figure 3). By restricted cubic splines (RCS), as shown in Figure 4, we found a positive correlation between ln-HbEO and ln-sNfL levels after adjusting for all covariates (*p* for nonlinear = 0.1249).

**Table 2:** The association between EO exposure (continuous variables) and serum NfL levels.

ln-HbEO (pmol/g Hb)	*β* (SE)	95% CI^a^		*p*-value
		Lower	Upper	
Crude model	0.06 (0.027)	0.00	0.12	0.036
Model 1^b^	0.08 (0.028)	0.01	0.15	0.032
Model 2^c^	0.07 (0.025)	0.00	0.14	0.044

a CI, Confidence Interval.

b Adjusted for gender, age, ethnicity, annual household income, education level.

c Further adjusted for alcohol consumption and serum cotinine based on model 1.

**Table 3:** The association between EO exposure (categorical variables) and serum NfL levels.

ln-HbEO (pmol/g Hb)	*β* (SE)	95% CI^a^		*p*-value
		Lower	Upper	
Crude model				
Q1 (1.76 ~ <2.74)	Reference			
Q2 (2.74 ~ <3.09)	0.01 (0.062)	−0.12	0.15	0.8
Q3 (3.09 ~ <3.92)	0.18 (0.101)	−0.03	0.4	0.092
Q4 (3.92 ~ 7.17)	0.21 (0.094)	0.01	0.42	0.043
Model 1^b^				
Q1 (1.76 ~ <2.74)	Reference			
Q2 (2.74 ~ <3.09)	0.03 (0.043)	−0.11	0.16	0.6
Q3 (3.09 ~ <3.92)	0.19 (0.072)	−0.04	0.42	0.076
Q4 (3.92 ~ 7.17)	0.25 (0.084)	−0.02	0.52	0.059
Model 2^c^				
Q1^d^ (1.76 ~ <2.74)	Reference			
Q2 (2.74 ~ <3.09)	0.02 (0.043)	−0.11	0.16	0.6
Q3 (3.09 ~ <3.92)	0.21 (0.071)	−0.04	0.46	0.074
Q4 (3.92 ~ 7.17)	0.25 (0.095)	0.03	0.46	0.038

a CI, Confidence Interval.

b Adjusted for gender, age, ethnicity, annual household income, education level.

c Further adjusted for alcohol consumption and serum cotinine based on model 1.

d Q1, <2.74 pmol/g Hb; Q2, 2.74 ~ <3.09 pmol/g Hb; Q3, 3.09 ~ <3.92 pmol/g Hb; Q4, >3.92 pmol/g Hb.

### 3.3 Subgroup analysis

We further examined the association between ln-HbEO and ln-sNfL across different subgroups. As shown in Table 4, although the interaction effects were not statistically significant (*p* for interaction >0.05), several important patterns emerged, particularly across ethnic groups and age categories. A significant positive correlation was observed between ln-HbEO and ln-sNfL in the 40–59 age group (*β* = 0.06, 95% CI: 0.00–0.12), while the older age group (≥ 60 years) exhibited a slightly stronger but non-significant association (*β* = 0.08, 95% CI: −0.02-0.18). Furthermore, the association between EO exposure and serum NfL levels was most pronounced in Non-Hispanic white people (*β* = 0.09, 95% CI: 0.02–0.15), who also exhibited the highest baseline serum NfL levels (13.46 pg/mL) among all ethnic groups. Additionally, the relationship between EO exposure and serum NfL levels was particularly strong among heavy drinkers (*β* = 0.10, 95% CI: 0.02–0.17), suggesting potential synergistic effects between alcohol consumption and EO exposure on neural damage.

**Table 4:** The association between EO exposure and serum NfL levels among different subgroups.

Subgroups	Population	*β* (95% CI^a^)	P for interaction
Gender			0.979
Male	277 (51%)	0.07 (0.02, 0.13)*	
Female	282 (49%)	0.08 (0.00, 0.15)*	
Age (years)			0.848
20–39	203 (39%)	0.06 (−0.04, 0.16)	
40–59	210 (38%)	0.06 (0.00, 0.12)*	
≧ 60	146 (23%)	0.08 (−0.02, 0.18)	
Ethnicity			0.744
Mexican American	82 (10%)	0.15 (−0.01, 0.32)	
Non-Hispanic black people	91 (11%)	0.03 (−0.08, 0.14)	
Non-Hispanic white people	268 (67%)	0.09 (0.02, 0.15)*	
Other Hispanic	56 (6.3%)	−0.01 (−0.13, 0.12)	
Others	62 (5.6%)	0.03 (−0.22, 0.27)	
Annual household income (dollars)			0.858
≦ 25,000	176 (22%)	0.08 (−0.01, 0.18)	
≧ 25,000	383 (78%)	0.07 (0.02, 0.12)*	
Alcohol consumption			0.417
No	133 (20%)	0.08 (−0.01, 0.16)	
Moderate	172 (32%)	0.02 (−0.05, 0.09)	
Heavy	254 (49%)	0.10 (0.02, 0.17)*	
Cotinine (ng/mL)			0.664
< 0.05	292 (53%)	0.12 (−0.08, 0.31)	
0.05–2.99	166 (30%)	0.10 (−0.32, 0.53)	
≧ 3.00	101 (16%)	0.00 (−0.10, 0.10)	

a CI, Confidence Interval.

### 3.4 Mediation analysis

**Figure 5:**
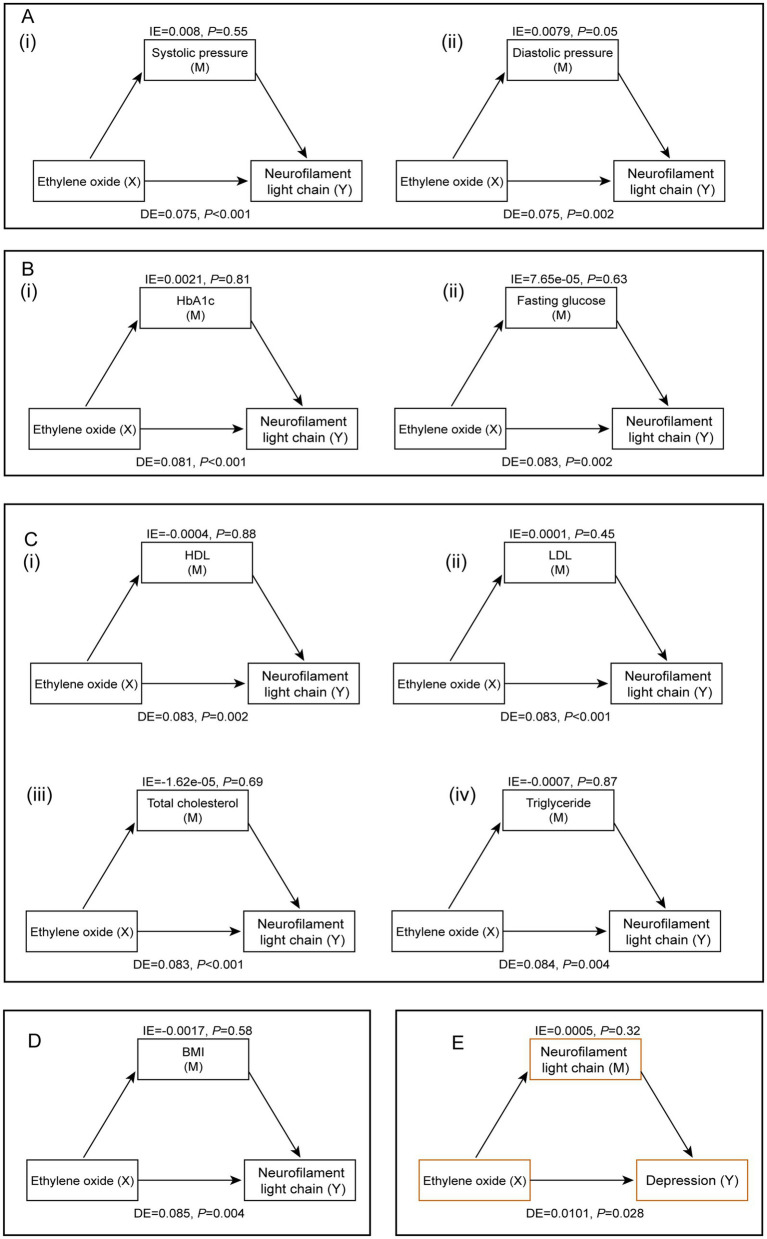
Mediation analysis of the relationship between EO exposure and serum NfL levels. **(A)** Blood pressure [(i) systolic pressure, (ii) diastolic pressure]. **(B)** Blood glucose [(i) HbA 1 c, (ii) fasting blood glucose]. **(C)** Blood lipids [(i) HDL, (ii) LDL, (iii) total cholesterol, (iv) triglycerides]. **(D)** Body mass index. **(E)** The potential mediating role of serum NfL levels in the relationship between EO and depression.

Figure 5 illustrated the mediating factors in the association between HbEO and serum NfL, as well as the potential mediating effect of serum NfL in the relationship between HbEO and depression. After adjusting for all covariates, we found no significant mediating effects of blood pressure (systolic and diastolic), blood glucose (HbA1c, fasting glucose), blood lipids (HDL, LDL, total cholesterol, triglycerides), or BMI. Additionally, mediation analysis also ruled out the mediating effect of serum NfL in the relationship between HbEO and depression. Finally, sensitivity analyses using different datasets emphasized the positive correlation between EO exposure and serum NfL levels, further strengthening the robustness of our findings (Supplementary Tables S1–S4).

## 4 Discussion

This study investigated the association between EO exposure and serum NfL levels in the adult population of the United States. Using data from NHANES 2013–2014, we found a positive correlation between EO exposure and levels of serum NfL, a sensitive biomarker of neural injury, in the serum. Our results indicated that as EO exposure levels increase, serum NfL levels also rise, suggesting a potential neurotoxic effect of EO. The strength of this study lied in its ability to generalize the findings to American adults aged 20 and older, due to the consideration of the complex sampling design. Moreover, the information provided by the NHANES database was reliable and comprehensive.

Current evidence linked environmental pollutants with biomarkers of neurodegenerative diseases. Some common compounds, such as volatile organic compounds (17) and Di (2-ethylhexyl) phthalate (24), have been shown to be positively correlated with higher serum NfL levels. At the same time, our findings are consistent with previous research emphasizing the neurotoxic potential of EO. Earlier studies have demonstrated that EO is a potent mutagen and carcinogen (2, 25). Occupational exposure to EO may increase the risk of mortality from malignant tumors of the lymphatic and hematopoietic systems (25, 26). Song et al. found a significant association between EO exposure and the risk of chronic non-obstructive pulmonary disease based on NHANES data (5). Additionally, adverse health outcomes such as cognitive impairment and memory loss have been reported among individuals exposed to EO (2). Another NHANES-based study reported a significant association between EO exposure and an increased risk of depression (11). Jiang et al. also found a significant “J”-shaped nonlinear dose–response relationship between HbEO levels and depression in a representative U.S. population (10). Building on these findings, this study demonstrated a statistically significant association between EO exposure and biomarkers of neural injury, suggesting that EO may contribute to neurodegenerative processes. Mechanistic insights from previous research supported the biological plausibility of EO’s neurotoxic effects. EO was known to alkylate DNA and proteins, leading to cellular damage and oxidative stress (27, 28). Neuronal axons, rich in neurofilament proteins such as NfL, are particularly sensitive to oxidative stress and toxic exposure (29). What’s more, NfL is a cytoskeletal protein in neuronal axons, and elevated serum NfL levels reflect axonal damage and neuronal loss, and it is a marker for various neurodegenerative diseases (30, 31). Therefore, the observed elevation in serum NfL levels in individuals with higher HbEO concentrations is biologically plausible. The observed correlation between EO exposure and increased serum NfL levels suggested that EO may cause axonal damage, further supporting its classification as a neurotoxic substance.

Our study results also indicated that HbEO and serum NfL levels can vary significantly based on demographic factors. In our research, males, older individuals, and non-Hispanic white people exhibited higher serum NfL concentrations, consistent with previous studies (12, 24). Concurrently, males, younger individuals, non-Hispanic black people, individuals with lower BMI, and those with lower education levels had higher HbEO levels, suggesting that different demographic backgrounds or socioeconomic statuses influenced EO exposure distribution levels. Additionally, we found that individuals with high serum cotinine levels had extremely high HbEO levels. Correspondingly, Zhu et al. (32) using NHANES representative data, confirmed a significant positive correlation between serum cotinine and serum NfL levels in adult subjects, highlighting the potential impact of smoking on neural function impairment. Furthermore, our subgroup analysis showed that the association between EO exposure and serum NfL levels was consistent across different demographic groups, including age, gender, race, and smoking status. However, we noted a significant positive correlation in specific subgroups, particularly among non-Hispanic white people, individuals aged 40–59, and heavy drinkers. These findings suggested that the neurotoxic effects of EO might be widespread across various subgroups, though certain populations may exhibit higher susceptibility. Further research is needed to understand the underlying mechanisms of this observed relationship.

Several cross-sectional studies based on NHANES data have confirmed the association between EO exposure and the risk of metabolic diseases such as obesity (33), diabetes (7), and hypertension (8). Additionally, studies have suggested that hypertension, diabetes, and dyslipidemia are significantly associated with elevated serum NfL levels (34, 35). Interestingly, however, our mediation analysis did not find significant mediating effects of blood pressure, blood glucose, lipids, and BMI on the relationship between HbEO and serum NfL levels. This indicated that the impact of EO exposure on NfL levels is not indirectly mediated through these traditional metabolic and lipid indicators but may be due to a direct effect or other important unconsidered mediators such as inflammatory factors or oxidative stress markers. Oxidative stress was a known pathway leading to neuronal damage and neurodegeneration (14). Chronic inflammation has been demonstrated to be associated with various neurodegenerative diseases (36), and EO exposure may trigger inflammatory responses (9), resulting in neuronal damage. Future research should explore these pathways to better understand the mechanisms by which EO exposure leads to increased serum NfL levels. Moreover, our findings indicated that serum NfL levels did not mediate the relationship between HbEO and depression, suggesting that EO’s neurotoxic effects may not indirectly translate into depressive symptoms through changes in serum NfL levels. Nevertheless, current studies have revealed an association between EO exposure and the risk of depression (10, 11), and high serum NfL levels also have been reported to be associated with more depressive symptoms (37, 38). Therefore, considering the sample size and biological differences among individuals, it remains necessary to continue designing rigorous studies to extensively explore the unclear mediation pathways.

The strengths of this study include the use of a large, nationally representative sample and the application of robust analytical methods. By employing multiple linear regression models and restricted cubic spline analysis, we comprehensively evaluated the relationship between EO exposure and serum NfL levels and investigated potential mediating factors. However, several limitations must be acknowledged. Firstly, the cross-sectional nature of NHANES data limited causal inference. Secondly, although HbEO is a well-validated biomarker of EO exposure, it might not capture all sources of EO exposure, such as short-term or low-level exposures. Thirdly, the potential residual confounding by unmeasured variables were not completely ruled out. Lastly, our findings may be specific to the U.S. population, necessitating comparisons with similar studies in other populations to validate our results. Future research should address these limitations by increasing the sample size, employing longitudinal study designs, and exploring other biomarkers of EO exposure and neurotoxicity. Additionally, investigating the mechanistic pathways through which EO induces neural damage will be crucial for understanding its neurotoxic potential.

In conclusion, this study provided a strong evidence of a positive correlation between EO exposure and serum NfL levels in a representative adult population in the United States, suggesting a potential neurotoxic effect of EO. Our findings highlighted the necessity for continued monitoring and regulation of EO exposure to mitigate its adverse health impacts. By emphasizing the utility of serum NfL as a biomarker for EO-induced neural damage, this study offered robust data supporting the neurotoxic effects of environmental pollutants and underscores the importance of protecting public health from harmful chemical exposures.

## Data Availability

The original contributions presented in the study are included in the article/Supplementary material, further inquiries can be directed to the corresponding authors.

## Footnotes

^1^ https://wwwn.cdc.gov/Nchs/Nhanes/2013-2014/ETHOX_H.htm

## References

[1] de Sousa Iwamoto LA Duailibi MT Iwamoto GY de Oliveira DC Duailibi SE. Evaluation of ethylene oxide, gamma radiation, dry heat and autoclave sterilization processes on extracellular matrix of biomaterial dental scaffolds. Sci Rep. (2022) 12:4299. doi: 10.1038/s41598-022-08258-1, PMID: 35277556 PMC8916068

[2] Lynch HN Kozal JS Russell AJ. Systematic review of the scientific evidence on ethylene oxide as a human carcinogen. Chem Biol Interact. (2022) 364:110031. doi: 10.1016/j.cbi.2022.110031 35779612

[3] Filser JGDB Törnqvist M Kessler W. Pharmacokinetics of ethylene in man; body burden with ethylene oxide and hydroxyethylation of hemoglobin due to endogenous and environmental ethylene. Arch Toxicol. (1992) 66:157–63. doi: 10.1007/BF01974008, PMID: 1303633

[4] Sheehan PJ Lewis RC Kirman CR Watson HN Winegar ED Bus JS. Ethylene oxide exposure in U.S. populations residing near sterilization and other industrial facilities: context based on endogenous and Total equivalent concentration exposures. Int J Environ Res Public Health. (2021) 18:607. doi: 10.3390/ijerph18020607, PMID: 33445726 PMC7828163

[5] Song W Hu H Ni J Zhang H Zhang H Yang G. The relationship between ethylene oxide levels in hemoglobin and the prevalence of kidney stones in US adults: an exposure–response analysis from NHANES 2013–2016. Environ Sci Pollut Res. (2022) 30:26357–66. doi: 10.1007/s11356-022-24086-2, PMID: 36367648

[6] Li Z Shi P Chen Z Zhang W Lin S Zheng T. The association between ethylene oxide exposure and asthma risk: a population-based study. Environ Sci Pollut Res. (2022) 30:24154–67. doi: 10.1007/s11356-022-23782-3 36334203

[7] Guo J Wan Z Cui G Pan A Liu G. Association of exposure to ethylene oxide with risk of diabetes mellitus: results from NHANES 2013–2016. Environ Sci Pollut Res. (2021) 28:68551–9. doi: 10.1007/s11356-021-15444-7, PMID: 34273079

[8] Wu N Cao W Wang Y Liu X. Association between blood ethylene oxide levels and the prevalence of hypertension. Environ Sci Pollut Res. (2022) 29:76937–43. doi: 10.1007/s11356-022-21130-z, PMID: 35668269

[9] Zhu X Kong X Chen M. Blood ethylene oxide, systemic inflammation, and serum lipid profiles: results from NHANES 2013–2016. Chemosphere. (2022) 299:134336. doi: 10.1016/j.chemosphere.2022.134336, PMID: 35337822

[10] Jiang S Wang Y Wang M Xu Y Zhang W Zhou X. Sex difference in the non-linear relationship between ethylene oxide exposure and depressive symptoms: a cross-sectional study. J Affect Disord. (2024) 345:386–93. doi: 10.1016/j.jad.2023.10.147, PMID: 37918573

[11] Wang H Chen X Lin F Zheng J Chen K Wang X. Association between ethylene oxide levels and depressive symptoms: a cross-sectional study based on NHANES 2013–2018 database. J Affect Disord. (2024) 348:135–42. doi: 10.1016/j.jad.2023.12.050, PMID: 38154580

[12] Beltran TA. Normative values for serum Neurofilament light chain in US adults. J Clin Neurol. (2024) 20:46–9. doi: 10.3988/jcn.2022.0340, PMID: 38179631 PMC10782095

[13] Silvestro S Raffaele I Quartarone A Mazzon E. Innovative insights into traumatic brain injuries: biomarkers and new pharmacological targets. Int J Mol Sci. (2024) 25:372. doi: 10.3390/ijms25042372, PMID: 38397046 PMC10889179

[14] Burgetova A Dusek P Uher T Vaneckova M Vejrazka M Burgetova R. CSF markers of oxidative stress are associated with brain atrophy and Iron accumulation in a 2-year longitudinal cohort of early MS. Int J Mol Sci. (2023) 24:10048. doi: 10.3390/ijms241210048, PMID: 37373196 PMC10298232

[15] Aamodt WW Waligorska T Shen J Tropea TF Siderowf A Weintraub D. Neurofilament light chain as a biomarker for cognitive decline in Parkinson disease. Mov Disord. (2021) 36:2945–50. doi: 10.1002/mds.28779, PMID: 34480363 PMC8688198

[16] Gaetani L Blennow K Calabresi P di Filippo M Parnetti L Zetterberg H. Neurofilament light chain as a biomarker in neurological disorders journal of neurology. J Neurol Neurosurg Psychiatry. (2019) 90:870–81. doi: 10.1136/jnnp-2018-320106, PMID: 30967444

[17] Bi Z Meng Y Ji Q. Association between volatile organic compounds and serum neurofilament light chain in US adults. Sci Total Environ. (2024) 926:171893. doi: 10.1016/j.scitotenv.2024.171893 38531449

[18] Yang A-M Chu P-L Wang C Lin CY. Association between urinary glyphosate levels and serum neurofilament light chain in a representative sample of US adults: NHANES 2013–2014. J Expo Sci Environ Epidemiol. (2023) 34:287–93. doi: 10.1038/s41370-023-00594-2 37674008

[19] Ogawa MOT Isse T Yamaguchi T Murakami T Endo Y Kawamoto T. Hemoglobin adducts as a marker of exposure to chemical substances, especially PRTR class I designated chemical substances. J Occup Health. (2006) 48:314–28. doi: 10.1539/joh.48.314, PMID: 17053297

[20] Li H-r Fu X-h Song L-l. Association between pyrethroid exposure and risk of depressive symptoms in the general US adults. Environ Sci Pollut Res. (2022) 30:685–98. doi: 10.1007/s11356-022-22203-9, PMID: 35904735

[21] Jain RB. Revised and extended serum cotinine cut-offs to classify smokers and non-smokers. Biomarkers. (2018) 23:502–7. doi: 10.1080/1354750x.2018.1443516, PMID: 29465001

[22] Jain RB. Associations between observed concentrations of ethylene oxide in whole blood and smoking, exposure to environmental tobacco smoke, and cancers including breast cancer: data for US children, adolescents, and adults. Environ Sci Pollut Res. (2020) 27:20912–9. doi: 10.1007/s11356-020-08564-z, PMID: 32249385

[23] Wu Y Song J Zhang Q. Association between organophosphorus pesticide exposure and depression risk in adults: a cross-sectional study with NHANES data. Environ Pollut. (2023) 316:120445. doi: 10.1016/j.envpol.2022.120445, PMID: 36265728

[24] Yang A-M Lai T-S Lin Y-L Wang CK Lin CY. Urinary di-(2-ethylhexyl) phthalate metabolites are independently related to serum neurofilament light chain, a biomarker of neurological diseases, in adults: results from NHANES 2013–2014. Environ Sci Pollut Res. (2023) 30:66417–25. doi: 10.1007/s11356-023-26943-0, PMID: 37097562

[25] Stayner LSK Greife A Hornung R Hayes RB Nowlin S Morawetz J. Exposure-response analysis of Cancer mortality in a cohort of workers exposed to ethylene oxide. Am J Epidemiol. (1993) 138:787–98. doi: 10.1093/oxfordjournals.aje.a116782, PMID: 8237967

[26] Jones RR Fisher JA Medgyesi DN Buller ID Liao LM Gierach G. Ethylene oxide emissions and incident breast cancer and non-Hodgkin lymphoma in a US cohort. JNCI J Natl Cancer Inst. (2023) 115:405–12. doi: 10.1093/jnci/djad004, PMID: 36633307 PMC10086621

[27] Adedara IA Farombi EO. Induction of oxidative damage in the testes and spermatozoa and hematotoxicity in rats exposed to multiple doses of ethylene glycol monoethyl ether. Hum Exp Toxicol. (2010) 29:801–12. doi: 10.1177/0960327109360115, PMID: 20172899

[28] Rasool M Malik A Abdul Basit Ashraf M Mubbin R Ayyaz U Waquar S. Phytochemical analysis and protective effects of *Vaccinium macrocarpon* (cranberry) in rats (*Rattus norvegicus*) following ethylene oxide-induced oxidative insult. Bioengineered. (2021) 12:4593–604. doi: 10.1080/21655979.2021.1955528, PMID: 34346287 PMC8806514

[29] Palmieri M Frati A Santoro A Frati P Fineschi V Pesce A. Diffuse axonal injury: clinical prognostic factors, molecular experimental models and the impact of the trauma related oxidative stress. An extensive review concerning milestones and advances. Int J Mol Sci. (2021) 22:10865. doi: 10.3390/ijms221910865, PMID: 34639206 PMC8509530

[30] Abu-Rumeileh S Abdelhak A Foschi M D'Anna L Russo M Steinacker P. The multifaceted role of neurofilament light chain protein in non-primary neurological diseases. Brain. (2023) 146:421–37. doi: 10.1093/brain/awac328, PMID: 36083979 PMC9494370

[31] Meier S Willemse EAJ Schaedelin S Oechtering J Lorscheider J Melie-Garcia L. Serum glial fibrillary acidic protein compared with Neurofilament light chain as a biomarker for disease progression in multiple sclerosis. JAMA Neurol. (2023) 80:287–97. doi: 10.1001/jamaneurol.2022.5250, PMID: 36745446 PMC10011932

[32] Zhu N Zhu J Lin S Yu H Cao C. Correlation analysis between smoke exposure and serum neurofilament light chain in adults: a cross-sectional study. BMC Public Health. (2024) 24:24. doi: 10.1186/s12889-024-17811-8, PMID: 38308244 PMC10835908

[33] Zhou C Wang S Ju L Zhang R Yang Y Liu Y. Positive association between blood ethylene oxide levels and metabolic syndrome: NHANES 2013-2020. Front Endocrinol. (2024) 15:15. doi: 10.3389/fendo.2024.1365658, PMID: 38699390 PMC11063307

[34] Korley FK Goldstick J Mastali M van Eyk JE Barsan W Meurer WJ. Serum NfL (Neurofilament light chain) levels and incident stroke in adults with diabetes mellitus. Stroke. (2019) 50:1669–75. doi: 10.1161/strokeaha.119.024941, PMID: 31138085 PMC6591022

[35] O'Bryant S Petersen M Hall J Johnson L Yaffe K Braskie M. Characterizing plasma NfL in a community-dwelling multi-ethnic cohort: results from the HABLE study. Alzheimers Dement. (2021) 18:240–50. doi: 10.1002/alz.12404, PMID: 34310015 PMC9228481

[36] Kwon HS Koh S-H. Neuroinflammation in neurodegenerative disorders: the roles of microglia and astrocytes. Transl Neurodegener. (2020) 9:42. doi: 10.1186/s40035-020-00221-2, PMID: 33239064 PMC7689983

[37] Chen M-H Liu Y-L Kuo H-W Tsai SJ Hsu JW Huang KL. Neurofilament light chain is a novel biomarker for major depression and related executive dysfunction. Int J Neuropsychopharmacol. (2022) 25:99–105. doi: 10.1093/ijnp/pyab068, PMID: 34637515 PMC8832224

[38] Schuurmans IK Ghanbari M Cecil CAM Ikram MA Luik AI. Plasma neurofilament light chain in association to late-life depression in the general population. Psychiatry Clin Neurosci. (2023) 78:97–103. doi: 10.1111/pcn.13608, PMID: 37843431

[39] ATSDR. Registry ATSDR clinician brief ethylene oxide. Atlanta, GA: U.S. Public Health Service, U.S. Department of Health and Human Services (2024).

