# Supplementary material for: Association of ethylene oxide exposure with serum neurofilament light chain levels among American adults
Source: Front Public Health. 2025 Mar 12;13:1545164. doi: 10.3389/fpubh.2025.1545164 (PMC11938061; doi:10.3389/fpubh.2025.1545164)
Supplement: Supplementary file 1 [file Table_1.docx]

**Supplementary files**

**Table S1. Sensitive analysis 1: The association between EO exposure (continuous variables) and serum NfL levels (n=641).**

| **ln-HbEO (pmol/g Hb)** | **β (SE)** | **95%CI^a^** | | ***P*-value** |
| --- | --- | --- | --- | --- |
|  |  | **Lower** | **Upper** |  |
| Crude model | 0.06 (0.029) | 0.00 | 0.13 | 0.039 |
| Model 1^b^ | 0.08 (0.028) | 0.01 | 0.15 | 0.038 |
| Model 2^c^ | 0.07 (0.027) | 0.01 | 0.15 | 0.028 |
| ^a^ CI = Confidence Interval. | | | | |
| ^b^ Adjusted for gender, age, ethnicity, annual household income, education level. | | | | |
| ^c^ Further adjusted for alcohol consumption and serum cotinine based on model 1. | | | | |

**Table S2. Sensitive analysis 1: The association between EO exposure (categorical variables) and serum NfL levels (n=641).**

| **ln-HbEO (pmol/g Hb)** | **β (SE)** | **95%CI^a^** | | *P*-value |
| --- | --- | --- | --- | --- |
|  |  | **Lower** | **Upper** |  |
| Crude model |  |  |  |  |
| Q1 (1.76~ <2.74) | *Reference* |  |  |  |
| Q2 (2.74~ <3.09) | 0.01 (0.055) | -0.11 | 0.13 | 0.8 |
| Q3 (3.09~ <3.94) | 0.19 (0.096) | -0.02 | 0.4 | 0.071 |
| Q4 (3.94~ 7.17) | 0.21 (0.090) | 0.01 | 0.41 | 0.038 |
| Model 1^b^ |  |  |  |  |
| Q1 (1.76~ <2.74) | *Reference* |  |  |  |
| Q2 (2.74~ <3.09) | 0.02 (0.037) | -0.1 | 0.14 | 0.7 |
| Q3 (3.09~ <3.94) | 0.20 (0.070) | -0.03 | 0.42 | 0.067 |
| Q4 (3.94~ 7.17) | 0.24 (0.080) | -0.01 | 0.5 | 0.057 |
| Model 2^c^ |  |  |  |  |
| Q1^d^ (1.76~ <2.74) | *Reference* |  |  |  |
| Q2 (2.74~ <3.09) | 0.01 (0.037) | -0.11 | 0.13 | 0.8 |
| Q3 (3.09~ <3.94) | 0.21 (0.075) | -0.03 | 0.45 | 0.071 |
| Q4 (3.94~ 7.17) | 0.25 (0.073) | 0.02 | 0.49 | 0.039 |
| ^a^ CI = Confidence Interval. | | | | |
| ^b^ Adjusted for gender, age, ethnicity, annual household income, education level. | | | | |
| ^c^ Further adjusted for alcohol consumption and serum cotinine based on model 1. | | | | |
| ^d^ Q1, <2.74 pmol/g Hb; Q2, 2.74~ <3.09 pmol/g Hb; Q3, 3.09~ <3.94 pmol/g Hb; Q4, >3.94 pmol/g Hb. | | | | |

**Table S3. Sensitive analysis 2: The association between EO exposure (continuous variables) and serum NfL levels (n=559, unweighted).**

| **ln-HbEO (pmol/g Hb)** | **β (SE)** | **95%CI^a^** | | ***P*-value** |
| --- | --- | --- | --- | --- |
|  |  | **Lower** | **Upper** |  |
| Crude model | 0.05 (0.026) | 0.00 | 0.11 | 0.035 |
| Model 1^b^ | 0.05 (0.023) | 0.01 | 0.1 | 0.017 |
| Model 2^c^ | 0.06 (0.028) | 0.01 | 0.12 | 0.028 |
| ^a^ CI = Confidence Interval. | | | | |
| ^b^ Adjusted for gender, age, ethnicity, annual household income, education level. | | | | |
| ^c^ Further adjusted for alcohol consumption and serum cotinine based on model 1. | | | | |

**Table S4. Sensitive analysis 2: The association between EO exposure (categorical variables) and serum NfL levels (n=559, unweighted).**

| **ln-HbEO (pmol/g Hb)** | **β (SE)** | **95%CI^a^** | | *P*-value |
| --- | --- | --- | --- | --- |
|  |  | **Lower** | **Upper** |  |
| Crude model |  |  |  |  |
| Q1 (1.76~ <2.74) | *Reference* |  |  |  |
| Q2 (2.74~ <3.09) | -0.01 (0.080) | -0.17 | 0.15 | 0.9 |
| Q3 (3.09~ <3.92) | 0.13 (0.080) | -0.03 | 0.29 | 0.1 |
| Q4 (3.92~ 7.17) | 0.15 (0.080) | -0.01 | 0.30 | 0.066 |
| Model 1^b^ |  |  |  |  |
| Q1 (1.76~ <2.74) | *Reference* |  |  |  |
| Q2 (2.74~ <3.09) | 0.05 (0.066) | -0.08 | 0.18 | 0.4 |
| Q3 (3.09~ <3.92) | 0.15 (0.066) | 0.02 | 0.28 | 0.021 |
| Q4 (3.92~ 7.17) | 0.17 (0.070) | 0.04 | 0.31 | 0.014 |
| Model 2^c^ |  |  |  |  |
| Q1^d^ (1.76~ <2.74) | *Reference* |  |  |  |
| Q2 (2.74~ <3.09) | 0.04 (0.066) | -0.09 | 0.18 | 0.5 |
| Q3 (3.09~ <3.92) | 0.16 (0.066) | 0.03 | 0.29 | 0.018 |
| Q4 (3.92~ 7.17) | 0.16 (0.079) | 0.00 | 0.31 | 0.047 |
| ^a^ CI = Confidence Interval. | | | | |
| ^b^ Adjusted for gender, age, ethnicity, annual household income, education level. | | | | |
| ^c^ Further adjusted for alcohol consumption and serum cotinine based on model 1. | | | | |
| ^d^ Q1, <2.74 pmol/g Hb; Q2, 2.74~ <3.09 pmol/g Hb; Q3, 3.09~ <3.92 pmol/g Hb; Q4, >3.92 pmol/g Hb. | | | | |
